# Application of deep learning–based image reconstruction in MR imaging of the shoulder joint to improve image quality and reduce scan time

**DOI:** 10.1007/s00330-022-09151-1

**Published:** 2022-09-27

**Authors:** Malwina Kaniewska, Eva Deininger-Czermak, Jonas M. Getzmann, Xinzeng Wang, Maelene Lohezic, Roman Guggenberger

**Affiliations:** 1 Institute of Diagnostic and Interventional Radiology, University Hospital Zurich (USZ), Raemistrasse 100, CH-8091 Zurich, Switzerland; 2 University of Zurich (UZH), Raemistrasse 100, CH-8091 Zurich, Switzerland; 3 Department of Forensic Medicine and Imaging, Institute of Forensic Medicine, University of Zurich, Zurich, Switzerland; 4 Global MR Applications & Workflow, GE Healthcare, Houston, TX USA; 5 Applications & Workflow, GE Healthcare, Manchester, UK

**Keywords:** Magnetic resonance imaging, Deep learning, Convolutional neural network, Shoulder joint, Radial k-space acquisition

## Abstract

**Objectives:**

To compare the image quality and diagnostic performance of conventional motion-corrected periodically rotated overlapping parallel line with enhanced reconstruction (PROPELLER) MRI sequences with post-processed PROPELLER MRI sequences using deep learning-based (DL) reconstructions.

**Methods:**

In this prospective study of 30 patients, conventional (19 min 18 s) and accelerated MRI sequences (7 min 16 s) using the PROPELLER technique were acquired. Accelerated sequences were post-processed using DL. The image quality and diagnostic confidence were qualitatively assessed by 2 readers using a 5-point Likert scale. Analysis of the pathological findings of cartilage, rotator cuff tendons and muscles, glenoid labrum and subacromial bursa was performed. Inter-reader agreement was calculated using Cohen’s kappa statistic. Quantitative evaluation of image quality was measured using the signal-to-noise ratio (SNR) and contrast-to-noise ratio (CNR).

**Results:**

Mean image quality and diagnostic confidence in evaluation of all shoulder structures were higher in DL sequences (*p* value = 0.01). Inter-reader agreement ranged between kappa values of 0.155 (assessment of the bursa) and 0.947 (assessment of the rotator cuff muscles). In 17 cases, thickening of the subacromial bursa of more than 2 mm was only visible in DL sequences. The pathologies of the other structures could be properly evaluated by conventional and DL sequences. Mean SNR (*p* value = 0.01) and CNR (*p* value = 0.02) were significantly higher for DL sequences.

**Conclusions:**

The accelerated PROPELLER sequences with DL post-processing showed superior image quality and higher diagnostic confidence compared to the conventional PROPELLER sequences. Subacromial bursa can be thoroughly assessed in DL sequences, while the other structures of the shoulder joint can be assessed in conventional and DL sequences with a good agreement between sequences.

**Key Points:**

*• MRI of the shoulder requires long scan times and can be hampered by motion artifacts.*

*• Deep learning–based convolutional neural networks are used to reduce image noise and scan time while maintaining optimal image quality. The radial k-space acquisition technique (PROPELLER) can reduce the scan time and has potential to reduce motion artifacts.*

*• DL sequences show a higher diagnostic confidence than conventional sequences and therefore are preferred for assessment of the subacromial bursa, while conventional and DL sequences show comparable performance in the evaluation of the shoulder joint.*

**Supplementary Information:**

The online version contains supplementary material available at 10.1007/s00330-022-09151-1.

## Introduction

Magnetic resonance imaging (MRI) offers optimal bone and soft tissue contrast and is hence the preferred modality for the assessment of the shoulder joint [1, 2]. MRI allows the assessment in detail of different anatomic structures such as rotator cuff tendons, biceps tendon, labrum, and cartilage as well as respective pathologies with high accuracy [3–6].

Conventional MRI of the shoulder joint is usually performed using multiplanar fast spin-echo (FSE) sequences, resulting in acquisition of high-resolution images in different contrast weightings [7, 8]. However, motion artifacts due to the strenuous breathing of patients with multiple chronic conditions or from the pulsation of the neighbouring vessels are possible limitations that may lower the image quality of FSE sequences [9, 10]. As the shoulder is located peripherally in the body, problems related to patient positioning may occur. It may result in motion artifacts even in compliant patients, who present with a painful shoulder [11, 12].

To overcome these limitations, different reconstruction techniques to reduce motion artifacts have been proposed, e.g. radial k-space sampling by periodically rotated overlapping parallel lines with enhanced reconstruction (PROPELLER), also termed BLADE (Siemens) and MultiVane-XD (Philips) [13–15]. The PROPELLER technique collects data in concentrical parallel lines rotated around the k-space, which enables correction of spatial variations and eventually reduction of motion artifacts. The main drawback of the PROPELLER method is usually an increase in acquisition time [16].

Deep learning–based convolutional neural networks (DL) have been recently introduced to accelerate image reconstruction of conventional sequences, as they allow the reduction of image noise and scan time while maintaining optimal image contrast [17, 18]. Most MRI protocols with deep learning–based reconstructions routinely use FSE sequences, and their successful implementation for assessment of different musculoskeletal structures has been shown in various studies [19–21]; however, the application of the deep-learning reconstruction to the PROPELLER sequences has not been examined yet.

Combining the PROPELLER acquisition technique with DL image reconstruction could allow the suppression of motion artifacts and reduce image noise and scan time at the same time.

The aim of this study was to compare the image quality and diagnostic performance of conventional PROPELLER MRI sequences with those of accelerated PROPELLER MRI sequences after post-processing using a DL for the assessment of the shoulder joint.

## Materials and methods

Ethical board approval was obtained for this prospective cohort study. Written informed consent was obtained from each included patient.

### Patients

Patients with an indication for an MRI of the shoulder between June and October 2021 were prospectively scanned and included in the study. All sequences, including conventional sequences, were acquired in the coronal oblique, sagittal oblique and axial plane using the PROPELLER technique and then transferred to the viewer for the purpose of DL-based post-processing.

An a priori power analysis was performed to evaluate the minimum cohort size with an effect size of 0.5, an alpha error of 0.05, and a beta error of 0.2. There was a Laplace distribution of the data, resulting in a minimum cohort size of 23 patients. Therefore, we set our goal for the cohort size over the required 23 patients and finally included 30 patients.

**Fig. 1:**
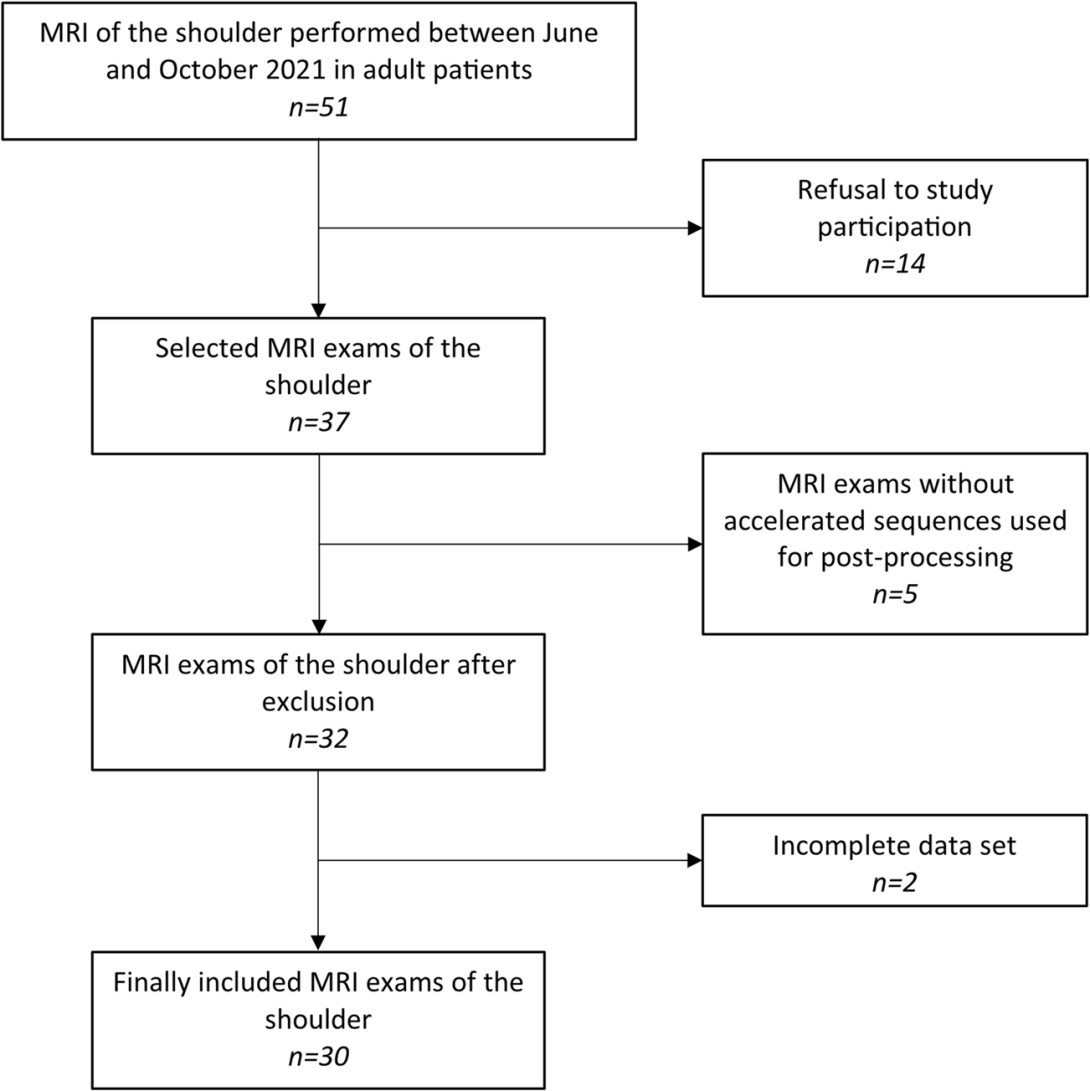
Flow diagram of patient selection

A total of 30 patients between 18 and 80 years of age with a male predominance (male *n* = 19, female *n* = 11) were included in the study. A flow chart with a detailed description is shown in Fig. 1.

### MRI protocol and MR acquisition

The MRI examinations were performed using a 1.5-T MRI scanner (SIGNA Artist, GE Healthcare) with a dedicated 16-channel shoulder coil.

The standard examination of the shoulder joint includes axial proton density (PD) fat-saturated (FS), sagittal oblique T2-weighted FS, sagittal oblique T1-weighted, coronal oblique PD FS, and coronal oblique T1-weighted FS sequences. The same sequences were additionally acquired with markedly reduced scan time but increased image noise and were subjected to a DL reconstruction algorithm (AIR^TM^ Recon DL®, GE Healthcare). All sequences were acquired using the PROPELLER technique.

The mean scan time of the standard protocol was 19 min 18 s, compared to 7 min 16 s for the accelerated protocol used for the DL-based reconstruction. For detailed MRI parameters, refer to Table 1.

**Table 1:** MRI parameters of conventional PROPELLER MRI sequences and accelerated PROPELLER MRI sequences used for post-processing

	Conventional PROPELLER sequences					Accelerated PROPELLER sequences used for post-processing				
	ax PD FS	sag T2w FS	sag T1w	cor PD FS	cor T1w FS	ax PD FS	sag T2w FS	sag T1w	cor PD FS	cor T1w FS
TE (ms)	60.8	97.1	604	61.6	19.3	72.1	102.5	23.0	58.7	22.9
TR (ms)	4541	6340	18.9	3562	593	5524	6828	681	3440	645
ST (mm)	3.5	3.5	3.5	3.5	3.5	3.2	3.5	3.5	3.5	3.5
Spacing between slices	3.8	3.8	3.8	3.8	3.8	3.5	3.8	3.8	3.8	3.8
Echo train length	18	28	6	18	6	22	30	7	18	7
Echo numbers	1	1	1	1	1	1	1	1	1	1
Matrix	288 × 288	280 × 280	288 × 288	288 × 288	288 × 288	340 × 340	320 × 320	320 × 320	340 × 340	320 × 320
Field of view (mm^2^)	150 × 150	150 × 150	150 × 150	150 × 150	150 × 150	150 × 150	150 × 150	150 × 150	150 × 150	150 × 150
Flip angle (°)	80	111	90	80	110	80	160	90	80	110
Receiver bandwidth (kHz)	162.7	162.7	195.3	162.7	195.3	195.3	244.1	244.1	244.1	244.1
Number of averages	3.3	3.25	2.0	3.4	2.5	1.8	1.8	1.6	1.8	1.6
Imaging frequency	63.8	63.8	63.8	63.8	63.8	63.8	63.8	63.8	63.8	63.8
Acquisition time (min:s)	04:33	04:17	03:36	03:34	03:18	02:10	01:40	01:47	01:50	01:49

*ax* axial, *cor* coronal oblique, *FOV* field of view, *FS* fat saturated, *PD* proton density, *sag* sagittal oblique, *ST* slice thickness, *T1w* T1-weighted, *T2w* T2-weighted, *TE* echo time, *TR* repetition time

Once raw accelerated MR images were acquired, they were transferred to the post-processing software (Orchestra SDK, GE Healthcare), and then reconstructed using the AIR^TM^ Recon DL algorithm and finally labelled as DL sequences.

The AIR^TM^ Recon DL pipeline includes a deep convolutional neural network (CNN) that operates on raw, complex-valued imaging data to produce a clean output image. Specifically, the CNN is designed to (1) provide a user-tuneable reduction in image noise, (2) reduce truncation artifacts, and (3) improve edge sharpness. Integration into the scanner’s native, inline reconstruction pipeline is critical as this provides access to raw, full bit-depth data. The CNN contains 4.4 million trainable parameters in approximately 10,000 kernels. It is a convolutional network, making it suitable for all MR relevant image sizes. The CNN was trained with a supervised learning approach using pairs of images representing near-perfect and conventional MRI images. The near-perfect training data consisted of high-resolution images with minimal ringing and very low noise levels. The conventional training data were synthesized from near-perfect images using established methods to create lower-resolution versions with more truncation artifacts and with higher noise levels [22].

The dedicated software for image post-processing (Orchestra SDK, GE Healthcare) uses the AIR^TM^ Recon DL algorithm to remove noise and Gibbs ringing artifacts from the raw data used as input before the final image is calculated [22]. Various levels of SNR improvement in the post-processing can be chosen between low, medium, and high. A maximum level of 100% for SNR improvement was used for DL sequences.

All image data sets were eventually sent to the PACS (IMPAX 6; Agfa-Gevaert N.V.) of our department for further analysis.

### Image analysis

In the initial training, a set of fifteen MRI examinations of the shoulder joint not included in the study sample was evaluated using conventional PROPELLER and post-processed DL sequences. Discrepancies were thoroughly discussed until the agreement was achieved. Then, MRI images were assessed independently by two readers (a board-certified radiologist with 6 years of experience and a board-certified radiologist with more than 15 years of experience in musculoskeletal radiology) blinded to any clinical information. All image sets have been stripped of all sequence identifiers (conventional sequence vs. DL sequences) and then mixed. The readers reviewed all images in a random order. After the readouts were performed, information on the sequence type was revealed for the purpose of the statistical analysis.

The intra-reader agreement was performed by the board-certified radiologist (M.K.) 8 weeks after the initial readout.

### Qualitative assessment of image quality

The image quality of conventional and DL sequences was assessed separately for bone and cartilage (humeral and glenoid), glenoid labrum, muscle (deltoid muscle and muscles of the rotator cuff), rotator cuff tendons, long head of biceps tendon, subcutaneous fat, and acromioclavicular joint using a 5-point Likert scale (0—poor, 1—mild, 2—moderate, 3—good, 4—perfect). The readers were instructed how to score the image quality basing on previously shown image examples illustrating each of the grade from the 5-point Likert scale. A score of 4 means the best image quality, with high image sharpness and no detectable image noise, and a perfect delineation of the analysed structures without any inhomogeneities or signal changes. A score of 3 was assigned to images comparable to the daily image quality, of a marginally inferior image quality with minor image noise, however with very good delineation of the analysed structures without notable inhomogeneities. A score of 2 was given to images of a considerably lower image quality with easily detectable image noise, with preserved delineation of the structures of the shoulder joint, with significant however not disturbing inhomogeneities. A score of 1 and 0 was given for images of poor image quality, with a lot of noise where delineation of the analysed structures was markedly distorted (score 1) or almost impossible (score 0).

Diagnostic confidence for evaluation of the above-mentioned structures together with assessment of contour sharpness and homogeneity of fat saturation in central and peripheral field of view (FOV) was performed using a 5-point scale (0—poor, 1—mild, 2—moderate, 3—good, 4—perfect). Readers rated diagnostic confidence as follows: score of 4: perfect lesion detection, a very high suspicion of a lesion; score of 3: good lesion detection, a high suspicion of a lesion; score of 2: lesion detection still possible, moderate suspicion of a lesion; score of 1: lesion detection hardly possible; and score of 0: inadequate assessment of any pathologies.

Central FOV was defined as the region of the glenohumeral joint at the level of midportion of glenoid, peripheral FOV as the most medial part of the pectoralis major muscle. Presence of motion artifacts was additionally evaluated in both image sets.

### Shoulder structures and associated pathologies

Evaluation of different joint structures was performed as follows: any pathological finding of the bone was noted and described. Cartilage was assessed as either (0) normal and homogenous, (1) focal areas of inhomogeneities with normal contour, (2) partial-thickness cartilage loss of less than 50%, or (3) partial-thickness cartilage loss of more than 50% or full-thickness cartilage loss with exposed subchondral bone. Muscle quality of the rotator cuff muscles was assessed as described by Goutallier et al [23, 24]. The quality of the supraspinatus (SSP) tendon was categorized as (0) normal, (1) tendinopathy, (2) articular-sided partial-thickness tear, (3) bursal-sided partial-thickness tear, and (4) full-thickness tear. The infraspinatus (ISP) and subscapularis (SSC) tendons were characterized as (0) normal, (1) tendinopathy, (2) partial-thickness tear, and (3) full-thickness tear. The quality and position of the long head of biceps tendon (LHBT) in the bicipital groove was evaluated as follows: (0) normal, (1) tendinopathy, (2) subluxation but still within the bicipital groove, and (3) displaced from the bicipital groove. The glenoid labrum was categorized as (0) normal, (1) mild, (2) moderate, (3) advanced degeneration, and (4) torn. The acromioclavicular (AC) joint was evaluated as (0) normal, (1) mild, (2) moderate, or (3) advanced degeneration. The subacromial bursa was characterized as (0) not visible; (1) less than 2 mm, considered normal; and (2) thickened over 2 mm, considered abnormal as described by White et al [25]. Each structure was assessed in all planes of the acquired image sets and sequences.

### Quantitative assessment of the image quality

To quantitatively assess image quality, the signal-to-noise ratio (SNR) and the contrast-to-noise ratio (CNR) for both sequences were measured. Regions of interest (ROIs) of 5 mm^2^ were placed separately on each set to define the signal intensity (SI) in bone (in the humeral head), muscle (deltoid muscle), and subcutaneous fat. The noise was defined as the standard deviation (SD) of the SI in a ROI measured in extracorporeal air.

The SNR and CNR were calculated as:

$$ {\displaystyle \begin{array}{c}\mathrm{SNR}=\frac{\mathrm{SI}}{\mathrm{SD}\left(\mathrm{air}\right)}\\ {}\mathrm{CNR}\left(\mathrm{bone}\right)=\frac{\mathrm{SI}\left(\mathrm{bone}\right)-\mathrm{SI}\left(\mathrm{muscle}\right)}{\mathrm{SD}\left(\mathrm{air}\right)}\\ {}\mathrm{CNR}\left(\mathrm{fat}\right)=\frac{\mathrm{SI}\left(\mathrm{fat}\right)-\mathrm{SI}\left(\mathrm{muscle}\right)}{\mathrm{SD}\left(\mathrm{air}\right)}\end{array}} $$

### Statistical analysis

All findings of image quality and diagnostic confidence were summarized and compared between conventional and DL sequences using a Wilcoxon signed-rank test [26]. Correlation between image quality and diagnostic confidence was calculated using Spearman rank correlation. A Shapiro-Wilk test was applied to assess the normal distribution of findings [27–29]. If a significant difference between sequences was noticed, a Bonferroni-Holm post hoc test for multiple comparison was additionally performed [30].

Agreement between conventional and DL sequences and inter-reader and intra-reader reliability for image quality and diagnostic confidence were calculated using the intraclass coefficient (ICC) [31]. ICC values under 0.5 were considered poor, between 0.5 and 0.75 moderate, between 0.75 and 0.9 good, and over 0.9 as an excellent reliability [32].

Pathologies of all the evaluated structures were recorded separately for each reader. Cohen’s kappa statistic was applied for inter-reader and intra-reader agreement for evaluation of pathological findings [33, 34]. Kappa values between 0.41 and 0.60 were considered moderate, between 0.61 and 0.80 substantial, and above 0.81 almost perfect agreement [35]. *p* < 0.05 was considered significant. All statistical analyses were conducted using SPSS, v. 26.0 (IBM).

## Results

In total, 30 patients (11 females, 19 males; age range 18–80 years) were included in the study. Patients’ characteristics (including age, gender, side of shoulder joint), indications for the shoulder MRI, and MRI findings are found in the Supplementary materials (Table 1.supp).

### Qualitative image quality and diagnostic confidence

The mean image quality of conventional and DL sequences in the assessment of bone was 2.6 and 3.8, respectively, for cartilage 2.2 and 3.6, for rotator cuff muscles 2.6 and 3.7, and for the glenoid labrum 2.6 and 3.5.

The mean diagnostic confidence for evaluation of bone was 3.5 and 3.8 for conventional and DL sequences, 3.6 and 3.9 for rotator cuff muscles, 2.8 and 3.7 for cartilage, and 2.9 and 3.6 for the glenoid labrum.

**Fig. 2:**
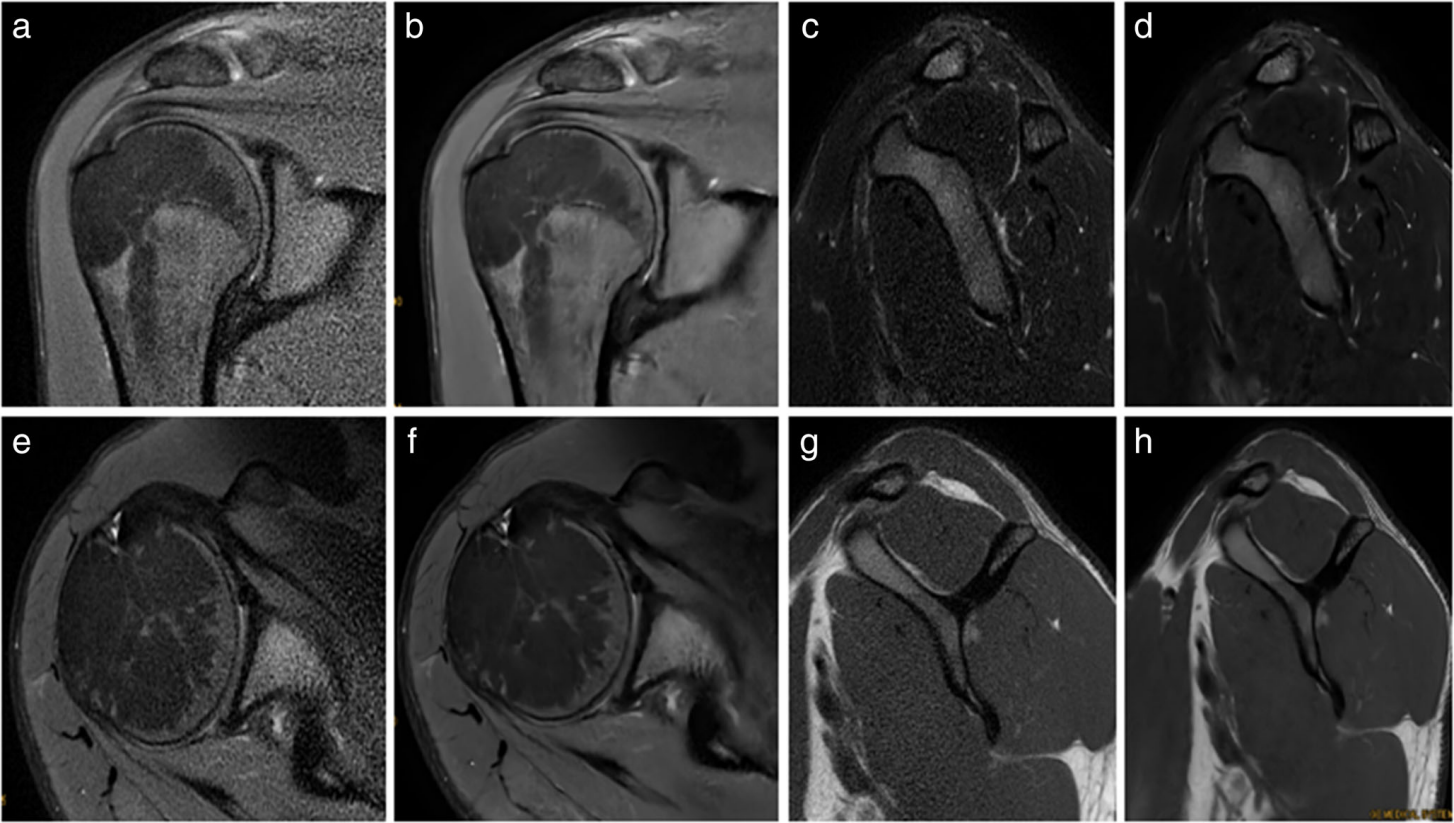
MR images of the right shoulder joint of a 32-year-old male. **a** Conventional PROPELLER coronal oblique proton density (PD) fat-saturated (FS) image, **b** coronal oblique PD FS image after post-processing using a deep learning–based convolutional neural network (DL), **c** conventional PROPELLER sagittal oblique T2-weighted (T2w) FS image, **d** sagittal oblique T2w FS image after post-processing using DL, **e** conventional PROPELLER axial PD FS image, **f** axial PD FS image after post-processing using DL, **g** conventional PROPELLER sagittal oblique T1-weighted (T1w) image, and **h** sagittal oblique T1w image after post-processing using DL

The mean image quality and diagnostic confidence were significantly better for DL sequences compared to conventional sequences for all analysed structures of the shoulder joint (*p* < 0.05)*.* Detailed information is found in Tables 2 and 3. Examples of both sequences are shown in Fig. 2.

**Table 2:** Image quality of all analysed structures of the shoulder joint in conventional and post-processed sequences using deep learning–based convolutional neural network (DL). For the assessment of the image quality of bone and cartilage bone glenoid and humeral were evaluated. Image quality of conventional and DL sequences was assessed using a 5-point Likert scale (0—poor, 1—mild, 2—moderate, 3—good, and 4—perfect)

	Reader 1		Reader 2		Wilcoxon signed-rank test (*p* value)
	Standard PROPELLER sequences (mean ± SD)	Post-processed PROPELLER sequencesusing DL (mean ± SD)	Standard PROPELLER sequences (mean ± SD)	PROPELLER sequences using DL (mean ± SD)	
Bone	2.6 ± 0.51	3.8 ± 0.31	2.5 ± 0.46	3.7 ± 0.37	
Cartilage	2.2 ± 0.56	3.6 ± 0.48	2.4 ± 0.61	3.4 ± 0.44	
Rotator cuff muscles	2.6 ± 0.72	3.7 ± 0.44	2.9 ± 0.63	3.6 ± 0.56	
Glenoid labrum	2.6 ± 0.72	3.5 ± 0.68	2.5 ± 0.62	3.7 ± 0.47	
Deltoid muscle	2.8 ± 0.42	3.7 ± 0.44	3.2 ± 0.67	3.7 ± 0.54	
Supraspinatus tendon	2.5 ± 0.62	3.9 ± 0.34	2.5 ± 0.73	3.6 ± 0.67	
Infraspinatus tendon	2.8 ± 0.76	3.7 ± 0.53	2.5 ± 0.77	3.7 ± 0.44	
Subscapularis tendon	2.6 ± 0.67	3.6 ± 0.56	2.7 ± 0.71	3.6 ± 0.49	
Long head of biceps tendon	2.8 ± 1.03	3.6 ± 0.97	2.9 ± 0.88	3.5 ± 0.81	
Acromioclavicular joint	2.2 ± 0.62	3.8 ± 0.37	2.8 ± 0.64	3.9 ± 0.30	
Subcutaneous fat tissue	3.1 ± 0.66	3.9 ± 0.34	2.8 ± 0.63	3.6 ± 0.56	All < 0.001

**Table 3:** Diagnostic confidence of all analysed structures of the shoulder joint in conventional and post-processed sequences using deep learning–based convolutional neural network (DL). Diagnostic confidence of conventional and DL sequences was assessed using a 5-point Likert scale (0—poor, 1—mild, 2—moderate, 3—good, and 4—perfect)

	Reader 1		Reader 2		Wilcoxon signed-rank test (*p* value)
	Conventional PROPELLER sequences (mean ± SD)	Post-processed PROPELLER sequences using DL (mean ± SD)	Conventional PROPELLER sequences (mean ± SD)	Post-processed PROPELLER sequences using DL (mean ± SD)	
Bone	3.5 ± 0.47	3.8 ± 0.44	3.6 ± 0.42	3.8 ± 0.38	< 0.001
Cartilage	2.8 ± 0.43	3.7 ± 0.45	2.9 ± 0.41	3.8 ± 0.46	< 0.001
Rotator cuff muscles	3.6 ± 0.47	3.9 ± 0.43	3.6 ± 0.56	3.8 ± 0.37	< 0.001
Glenoid labrum	2.9 ± 0.54	3.6 ± 0.47	3.2 ± 0.59	3.8 ± 0.40	< 0.001
Deltoid muscle	3.8 ± 0.37	3.9 ± 0.30	3.7 ± 0.52	3.8 ± 0.50	0.008
Supraspinatus tendon	3.4 ± 0.57	3.9 ± 0.43	3.4 ± 0.56	3.6 ± 0.72	< 0.001
Infraspinatus tendon	3.5 ± 0.56	3.8 ± 0.48	3.4 ± 0.62	3.8 ± 0.37	< 0.001
Subscapularis tendon	3.4 ± 0.61	3.7 ± 0.52	3.2 ± 0.43	3.6 ± 0.50	< 0.001
Long head of biceps tendon	3.3 ± 0.88	3.6 ± 0.96	3.4 ± 0.67	3.7 ± 0.78	< 0.001
Acromioclavicular joint	3.2 ± 0.58	3.8 ± 0.34	3.4 ± 0.57	3.8 ± 0.46	< 0.001
Subcutaneous fat tissue	3.8 ± 0.40	3.9 ± 0.43	3.5 ± 0.62	3.8 ± 0.46	0.001
Contour sharpness in central FOV	3.0 ± 0.41	3.9 ± 0.30	2.8 ± 0.37	3.8 ± 0.50	< 0.001
Homogeneity of fat saturation in central FOV	3.3 ± 0.47	3.8 ± 0.49	3.3 ± 0.75	3.6 ± 0.77	< 0.001
Contour sharpness in peripheral FOV	2.6 ± 0.62	3.3 ± 0.65	2.4 ± 0.71	3.1 ± 0.54	< 0.001
Homogeneity of fat saturation in peripheral FOV	2.7 ± 0.67	2.8 ± 0.54	2.6 ± 0.72	2.9 ± 0.49	< 0.001
Overall	3.0 ± 0.52	3.9 ± 0.34	3.2 ± 0.56	3.8 ± 0.50	< 0.05

### Assessment of shoulder structures and associated pathologies

In 17 cases, thickening of the subacromial bursa was identified using the DL sequences, while only in 7 cases using the conventional sequences with a significant difference between sequences in terms of proper delineation of the subacromial bursa (*p* < 0.05). The other analysed structures and associated pathological findings could be evaluated properly both by conventional and DL sequences.

**Fig. 3:**
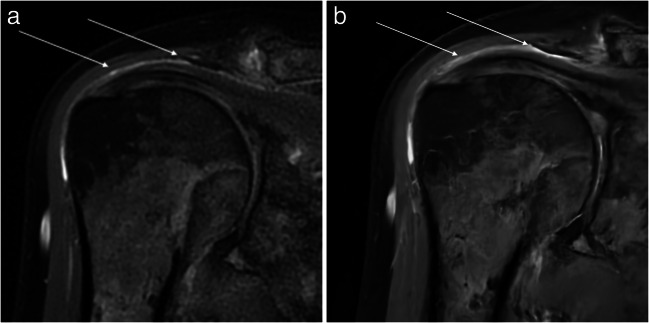
MR images of the right shoulder joint of a 71-year-old female. **a** Conventional PROPELLER coronal oblique proton density (PD) fat-saturated (FS) image, **b** coronal oblique PD FS image after post-processing using a deep learning–based convolutional neural network (DL) showing a subacromial bursa (arrow). The presence of a small amount of fluid within the subacromial bursa with a slight thickening of the subacromial bursa is clearly visible in DL sequences

**Fig. 4:**
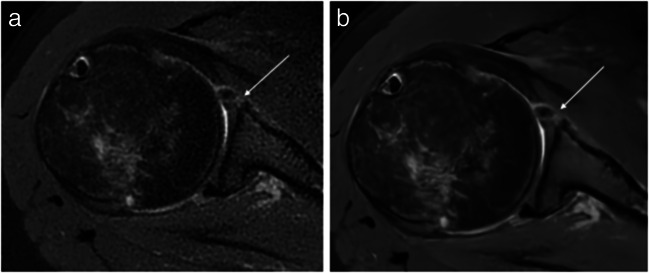
MR images of the right shoulder joint of a 40-year-old female with shoulder pain after anterior shoulder dislocation. **a** Conventional PROPELLER axial proton density (PD) fat-saturated (FS), **b** axial PD FS image after post-processing using DL shows a tear of the anterior midportion of the glenoid labrum (arrow). The pathology can be suggested both in conventional and post-processed MR sequences; however, it is more sharply delineated in the post-processed sequence

**Fig. 5:**
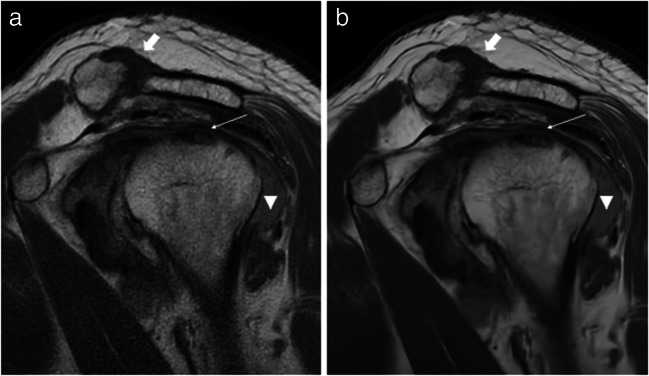
MR images of the right shoulder joint of a 78-year-old male with chronic shoulder pain. **a** Conventional PROPELLER sagittal oblique T1-weighted (T1w) image, **b** sagittal oblique T1w image after post-processing using DL images shows degenerative changes of the acromioclavicular joint (broad white arrow), subchondral cysts in the humeral head (thin white arrow), and a joint effusion (triangle). All pathologies can be delineated in both sequences; however, the post-processed sequence is less noisy so the pathologies can be identified easily

The summary of pathologies of all analysed structures in conventional and DL sequences as assessed by both readers can be found in Table 4. Examples of both sequences with pathological findings are shown in Figs. 3, 4 and 5.

**Table 4:** The summary of all analysed structures in conventional and post-processed sequences using deep learning–based convolutional neural network (DL)

	Pathological grading scale	Reader 1		Reader 2	
		Conventional PROPELLER sequences	Post-processed PROPELLER sequences using DL	Conventional PROPELLER sequences	Post-processed PROPELLER sequences using DL
Rotator cuff muscles	0 and 1. Normal	25	25	25	25
	2. Few fatty streaks	3	3	4	3
	3. Fat = muscle	1	1	0	1
	4. Fat > muscle	1	1	1	1
Cartilage	0. Normal and homogenous	4	10	12	10
	1. Focal areas of inhomogeneities with normal contour	21	15	13	15
	2. Partial-thickness cartilage loss of less than 50%	3	3	2	3
	3. Full-thickness cartilage loss with exposed subchondral bone	2	2	3	2
Glenoid labrum	0 and 1. Normal or mild changes	21	20	22	20
	2. Moderate degeneration	2	3	1	3
	3. Advanced degeneration	1	1	1	1
	4. Tear	6	6	6	6
Supraspinatus tendon	0. Normal	13	13	13	13
	1. Tendinopathy	6	6	8	6
	2. Articular-sided partial-thickness tear	6	6	4	6
	3. Bursal-sided partial-thickness tear	0	0	0	0
	4. Full-thickness tear	5	5	5	5
Infraspinatus tendon	0. Normal	21	21	23	21
	1. Tendinopathy	5	5	3	5
	2. Partial-thickness tear	1	1	1	1
	3. Full-thickness tear	3	3	3	3
Subscapularis tendon	0. Normal	5	6	11	6
	1. Tendinopathy	22	22	17	22
	2. Partial-thickness tear	1	1	1	1
	3. Full-thickness tear	2	1	1	1
Long head of biceps tendon	0. Normal	22	21	23	21
	1. Tendinopathy	3	3	2	3
	2. Subluxation but still within the bicipital groove	3	3	3	3
	3. Displaced from the bicipital groove	2	3	2	3
Acromioclavicular joint	0. Normal	5	5	5	5
	1. Mild degeneration	13	13	13	13
	2. Moderate degeneration	11	11	11	11
	3. Advanced degeneration	1	1	1	1
Subacromial bursa	0. Not visible	2	1	2	1
	1. Less than 2 mm	21	5	21	5
	2. Thickened over 2 mm	7	24	7	24

### Quantitative assessment of the image quality (SNR and CNR)

The mean SNR for bone, muscle, and fat was higher for DL sequences compared to conventional sequences with significant difference for muscle and fat (*p* < 0.05), but with no significant difference for bone (*p* > 0.05) (Table 7 and 8.supp)*.*

The mean CNR was significantly higher for DL sequences compared to conventional sequences (*p* < 0.05).

**Fig. 6:**
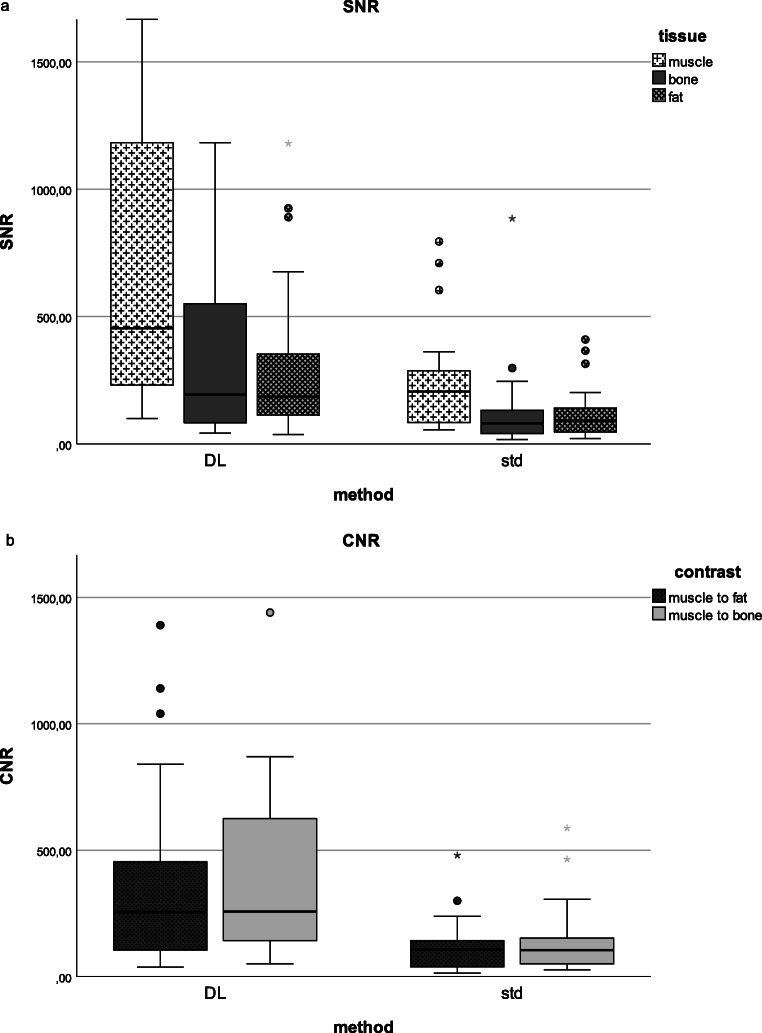
SNR (**a**) for bone, muscle, and fat and CNR (**b**) for post-processed sequences using DL and conventional sequences. Mean SNR for bone, muscle, and fat was higher for post-processed sequences using DL compared to conventional sequences with significant difference for muscle and fat (*p* < 0.05), but with no significant difference for bone*.* Mean CNR was significantly higher for post-processed sequences using DL compared to conventional sequences (*p* < 0.05)

Box plots for SNR and CNR are shown in Fig. 6. No motion artifacts were noted in any of the analysed image sets.

### Inter-reader agreement

There was a moderate overall inter-reader agreement for assessment of the image quality of conventional sequences with ICC values of 0.659 and 0.582 for DL sequences, respectively. There was a moderate inter-reader agreement for assessment of the diagnostic confidence of conventional and D sequences with ICC values of 0.695 and 0.595.

Detailed findings with inter-reader agreement for evaluation of image quality and diagnostic confidence are found in Supplementary materials (Table.supp 2 and 3).

In the evaluation of pathological findings, there was an almost perfect inter-reader agreement for evaluation of the rotator cuff muscles with kappa values of 0.947 and 0.892 for conventional and DL sequences, and a moderate agreement for assessment of the cartilage with kappa values of 0.524 and 0.532.

Inter-reader agreements for assessment of pathological findings are shown in Table 5.

**Table 5:** Inter-reader agreement for assessment of pathological findings of all investigated structures for conventional and post-processed sequence using deep learning convolutional neural network (DL). Inter-reader reliability for image quality and diagnostic confidence were calculated using intraclass coefficient (ICC). ICC values under 0.5 were considered poor, between 0.5 and 0.75 moderate, between 0.75 and 0.9 good, and over 0.9 as an excellent reliability

	Inter-reader agreement	
	Conventional PROPELLER sequences *K*-value	Post-processed PROPELLER sequences using DL *K*-value
Rotator cuff muscles	0.947	0.892
Cartilage	0.524	0.532
Supraspinatus tendon	0.811	0.904
Infraspinatus tendon	0.847	0.717
Subscapularis tendon	0.552	0.729
Long head of biceps tendon	0.684	0.587
Glenoid labrum	0.810	0.814
Acromioclavicular joint	0.663	0.659
Subacromial bursa	0.155	0.345

### Intra-reader agreement

There was a good overall intra-reader agreement for assessment of the image quality and diagnostic confidence of conventional and DL sequences with ICC values of 0.837 and 0.898 and 0.883 and 0.819, respectively.

There was an almost perfect intra-reader agreement for evaluation of the supraspinatus tendon and glenoid labrum of conventional and DL sequences with kappa values of 0.907 and 0.815 and 0.821 and 0.860, respectively. There was a substantial and almost perfect intra-reader agreement for assessment of the cartilage of conventional and DL sequences with kappa values of 0.741 and 0.841. Overall, there was a substantial intra-reader agreement for assessment of the pathological findings of all analysed structures with kappa values of 0.840 and 0.842 in conventional and DL sequences.

Intra-reader agreements for all analysed parameters are shown as Supplementary material (Table.supp 4-6).

## Discussion

To the best of our knowledge, this is the first study to combine the PROPELLER MR acquisition technique with a DL image reconstruction approach for imaging of the shoulder joint. The PROPELLER DL sequences showed substantially higher image quality of investigated anatomical structures of the shoulder joint compared to conventional PROPELLER sequences, resulting in higher diagnostic confidence and comparable diagnostic performance.

Dietrich et al described the use of the PROPELLER technique for MRI of the shoulder as a useful method for reduction of motion artifacts while increasing image quality [13]. The PROPELLER technique collects data in concentrical parallel lines rotated around the k-space, which enables correction of spatial variations and eventually reduction of motion artifacts [16]. The main drawback of the PROPELLER method is usually an increase in acquisition time. In our study, the mean acquisition time of the conventional PROPELLER sequences was 19 min 18 s and could be reduced to 7 min 16 s in the accelerated sequences used for post-processing using DL. This equals a reduction in scan time of 62%.

In conventional MR image reconstruction, suppression of Gibbs ringing artifacts results in a loss of spatial resolution and a lower image quality. With application of deep-learning-based vendor software used for the image post-processing, it is possible to suppress ringing artifacts while maintaining high image quality and resolution [22]. As expected, there was an overall better image quality and diagnostic confidence of DL sequences compared to conventional sequences. However, the delineation and detection of most pathological findings was equally possible using both sequences. The only exception was the subacromial bursa which could be better delineated and assessed in DL sequences. This may be explained by the higher image quality and subsequent easier delineation of subtle structures such as the subacromial bursa in DL sequences.

DL-based image reconstruction using FSE MR sequences has been applied for imaging of different organs including the brain, liver, heart, and peripheral nerves [21, 36–40]. Application of the PROPELLER technique with DL-based image reconstruction for imaging of the brain and prostate has been recently described and resulted in improvement of the SNR and image sharpness [41, 42].

The PROPELLER technique is a well-established method for image acquisition when motion reduction is desired especially in the imaging of the abdomen, lung, or shoulder joint [13, 16, 43–47]. Blood flow in the axillary vessels could be a potential source of pulsation artifacts in shoulder MRI; therefore, the use of the PROPELLER technique should be considered to minimize motion artifacts. Application of the PROPELLER technique in our study resulted in suppression of motion artifacts in both conventional and DL sequences, and no motion artifacts were noted.

These findings are in accordance with the study of Hahn et al who investigated the retrospective application of DL reconstructions for fast spin-echo sequences for accelerated shoulder MRI [19]. The mean scan time for accelerated MRI sequences in the study of Hahn et al was 3 min 5 s with the image quality lower than that in conventional MRI sequences, whereas application of deep-learning reconstruction resulted in image quality comparable with that of conventional sequences. While Hahn et al performed a retrospective study, we prospectively investigated a PROPELLER acquisition technique to minimize motion artifacts in combination with DL reconstruction. The substantial reduction of scan time not only allows for higher patient throughput per scanner but also likely affords higher patient comfort.

There was a moderate inter-reader agreement for image quality on conventional and DL sequences, and a moderate agreement for diagnostic confidence on both conventional and DL sequences. The unusual image impression of novel DL sequences to readers who were accustomed to reading conventional MR images might have impacted on subjective image quality perception.

Our study has several limitations. First, all MR images were acquired using the PROPELLER technique; hence, we did not perform a comparison of conventional FSE and the PROPELLER sequences for acquisition of the accelerated sequences used for post-processing using DL. While FSE sequences have been conventionally applied for acquisition of DL as previously described, there is lack of literature on application of the PROPELLER technique for deep-learning-based reconstructions [19–21].

Second, we did not follow up the patients with suspected injuries of the shoulder joint, and there was no correlation of MRI findings with an arthroscopic reference standard. Nevertheless, a good correlation of MR findings and arthroscopy in evaluation of the shoulder pathologies has been described in previous studies with a high accuracy in diagnosis of rotator cuff tears, osteochondral defects, and some labral tears, and in assessment of the muscle quality [48–54]. Moreover, the main aim of this study was to compare image quality and diagnostic performance of the conventional PROPELLER technique versus those using DL reconstructions. Finally, we did not analyse the impact of the PROPELLER technique on image quality and diagnostic performance for imaging of shoulder implants and postoperative susceptibility artifacts. This would be interesting to analyse in further studies.

In summary, the motion-corrected PROPELLER MR imaging technique with DL post-processing showed superior image quality and higher diagnostic confidence compared to the conventional PROPELLER sequences in imaging of the shoulder joint. Pathologies of the shoulder joint can be assessed correctly in the conventional PROPELLER and DL sequences. Due to significantly shorter scan times and higher SNR and CNR compared to conventional sequences, post-processed PROPELLER sequences using DL could be considered for clinical use after further validation at other sites.

## Supplementary Information

ESM 1 (DOCX 38 kb)

## Abbreviations

AC joint: Acromioclavicular joint
CNN: Convolutional neural network
CNR: Contrast-to-noise ratio
DL: Deep learning
FOV: Field of view
FSE: Fast spin-echo
ICC: Intraclass coefficient
ISP: Infraspinatus tendon
LHBT: Long head of biceps tendon
PROPELLER: Periodically rotated overlapping parallel lines with enhanced reconstruction
ROI: Regions of interest
SD: Standard deviation
SI: Signal intensity
SNR: Signal-to-noise ratio
SSC: Subscapularis tendon
SSP: Supraspinatus tendon

## Declarations

### Guarantor

The scientific guarantor of this publication is Roman Guggenberger.

### Statistics and biometry

No complex statistical methods were necessary for this paper.

### Methodology

• prospective

• diagnostic or prognostic study/observational

• performed at one institution
