# Supplementary material for: Application of deep learning–based image reconstruction in MR imaging of the shoulder joint to improve image quality and reduce scan time
Source: Eur Radiol. 2022 Sep 27;33(3):1513–25. doi: 10.1007/s00330-022-09151-1 (PMC9935676; doi:10.1007/s00330-022-09151-1)
Supplement: Supplementary file 1 — (DOCX 38 kb) [file 330_2022_9151_MOESM1_ESM.docx]

Supplementary table 1

| **Patient** | **Age** | **Gender** | **Shoulder side** | **Indication** | **Finding** |
| --- | --- | --- | --- | --- | --- |
| 1 | 79 | F | R | Shoulder pain, status post corticoid injection into subacromial bursa | Articular-sided partial-thickness tear of supraspinatus tendon, severe tendinopathy of the long biceps tendon. |
| 2 | 46 | F | R | Shoulder pain after a trauma; a positive biceps load test | Rotator cuff intact. Minor osteoarthritis of the AC joint. |
| 3 | 73 | M | L | Chronic shoulder pain | Dislocation of the AC joint, glenohumeral cartilage defects, mild labrum degeneration. |
| 4 | 49 | F | L | Chronic shoulder pain | Severe AC joint osteoarthritis. No relevant damage to the rotator cuff tendons. |
| 5 | 78 | M | R | Shoulder pain, especially at night | Severe shoulder osteoarthritis, severe labral degeneration, articular-sided partial-thickness tear of the supraspinatus and infraspinatus tendon |
| 6 | 54 | M | L | Reluctant shoulder pain after surgical refixation of the supraspinatus tendon | Recurrent full-thickness tear of supraspinatus tendon |
| 7 | 34 | M | L | Fall onto an outstretched hand | Full-thickness tear of the subscapularis tendon, anteroinferior labral tear with chondrolabral detachment and with intact attachment to the periosteum (Perthes lesion), acute Hill-Sachs lesion |
| 8 | 52 | M | L | Shoulder pain and limited range of motion | Diminished capacity of the axillary recess and interval in the MR arthrogram and thickening of the coracohumeral ligament consistent with adhesive capsulitis |
| 9 | 18 | M | R | Shoulder pain after a trauma; | Unremarkable MR exam of the shoulder joint |
| 10 | 56 | M | L | Reluctant shoulder pain; a positive biceps load test | SLAP lesion type 4 |
| 11 | 24 | M | R | Shoulder pain after a fall from a bicycle; no fracture in the initial X-ray | Unremarkable MR exam of the shoulder joint |
| 12 | 34 | F | R | Shoulder pain and limited range of motion | Moderate tendinopathy of the supraspinatus tendon with a small articular-sided partial tear posteriorly. Mild AC joint osteoarthritis. No evidence of adhesive capsulitis. |
| 13 | 26 | M | R | Shoulder pain after a motorbike accident | Acromioclavicular joint injury Rockwood type 2. |
| 14 | 52 | F | L | Persistent pain after rotator cuff repair | Superficial cartilage defects glenohumeral, no lesion of the rotator cuff |
| 15 | 66 | M | L | Recurrent shoulder pain; known tendinitis calcarea | Tendinopathy of the supraspinatus tendon, otherwise unremarkable rotator cuff. Narrowed acromiohumeral interval as a possible sign of subacromial impingement. |
| 16 | 57 | M | R | Chronic shoulder pain | Transmural full-thickness tear of the supraspinatus tendon with retraction of the tendon to the level of the humeral head (Patte Stage 2) |
| 17 | 24 | M | L | Status post traumatic anterior shoulder dislocation | Detachment of the anteroinferior labrum from the underlying glenoid (Bankart lesion) with extension superiorly, acute Hill-Sachs lesion. |
| 18 | 60 | M | R | Chronic shoulder pain | Transmural full-thickness tear of the supraspinatus and infraspinatus tendon with muscle atrophy and fatty degeneration (Goutallier grade 3). |
| 19 | 59 | M | R | Shoulder pain; known osseous and pulmonary metastatic acinar adenocarcinoma of the prostate | Multiple osseous metastases in the humerus, scapula and clavicle. No pathologic fracture. |
| 20 | 80 | F | L | Chronic shoulder pain | Transmural full-thickness tear of the supraspinatus and infraspinatus tendons with retraction of the tendon to the level of the glenoid. Muscle atrophy and fatty degeneration (Goutallier grade 3). Glenohumeral cartilage defects. |
| 21 | 42 | F | R | Shoulder pain and limited range of motion | Unremarkable MR exam of the shoulder joint |
| 22 | 40 | F | R | Traumatic anterior shoulder dislocation | Avulsion of the joint capsule and labrum from the anteroinferior glenoid rim (Bankart lesion), acute Hill-Sachs lesion. |
| 23 | 20 | M | R | Second traumatic anterior shoulder dislocation | Detachment of the anteroinferior labrum from the underlying glenoid (Bankart lesion), small acute Hill-Sachs lesion. |
| 24 | 25 | M | R | Shoulder pain after a fall from a bicycle; limited flexion and abduction due to pain. Function test not possible. No fracture in the initial X-ray. | Acromioclavicular joint injury Rockwood type 2. |
| 25 | 73 | F | R | Fall on right shoulder. Painful Arc. Limited elevation and abduction (below 90 degrees). X-ray calcification in the supraspinatus. | Extensive transmural rotator cuff tear and rupture of the long biceps tendon with retraction. Marked joint effusion with synovitis and bursitis subacromialis and subdeltoidea. |
| 26 | 38 | F | L | Fall onto an outstretched hand | Unremarkable MR exam of the shoulder joint. |
| 27 | 50 | M | R | Recurrent right shoulder pain. Positive biceps load test. Slight reduction in abduction strength. | Small articular-sided partial thickness tear of the supraspinatus tendon. Labrum degeneration. |
| 28 | 71 | F | R | Right shoulder pain. | Full-thickness tear of the supraspinatus tendon at the footprint with intramural tear in the retracted supraspinatus tendon. Tendinopathy of the subscapularis tendon. Ganglion in the spinoglenoid notch without neurogenic oedema or fatty degeneration of the supraspinatus and infraspinatus muscle. |
| 29 | 59 | M | R | Chronic shoulder pain | Severe osteoarthritis of the acromioclavicular joint. |
| 30 | 42 | M | R | Recurrent shoulder dislocations. | Fracture of the anteroinferior glenoid (bony Bankart lesion). Acute Hill-Sachs lesion. |

|  | **Conventional PROPELLER sequences**  **(ICC)** | **95% confidence interval** | **Post-processed PROPELLER sequences**  **using DL**  **(ICC)** | **95% confidence interval** |
| --- | --- | --- | --- | --- |
| Bone | 0.735 | 0.443 - 0.874 | 0.802 | 0.583 – 0.906 |
| Cartilage | 0.710 | 0.392 – 0.862 | 0.670 | 0.307 – 0.843 |
| Rotator cuff muscles | 0.788 | 0.555 – 0.899 | 0.712 | 0.528 – 0.732 |
| Glenoid labrum | 0.613 | 0.187 – 0.816 | 0.567 | 0.419 – 0.639 |
| Deltoid muscle | 0.715 | 0.401 – 0.864 | 0.783 | 0.506 – 0.812 |
| Supraspinatus tendon | 0.745 | 0.464 – 0.879 | 0.691 | 0.351 – 0.853 |
| Infraspinatus tendon | 0.531 | 0.016 – 0.777 | 0.883 | 0.754 – 0.944 |
| Subscapularis tendon | 0.764 | 0.504 – 0.888 | 0.746 | 0.467 – 0.879 |
| Long head of biceps tendon | 0.910 | 0.806 – 0.958 | 0.946 | 0.883 – 0.975 |
| Acromioclavicular joint | 0.369 | 0.211 – 0.399 | 0.415 | 0.321 – 0.645 |
| Subcutaneous fat tissue | 0.643 | 0.250 – 0.830 | 0.630 | 0.408 – 0.681 |
| Overall | 0.659 | 0.584 - 0.721 | 0.582 | 0.489 - 0.657 |

Supplementary table 2

|  | **Conventional PROPELLER sequences**  **(ICC)** | **95% confidence interval** | **Post-processed PROPELLER sequences**  **using DL**  **(ICC)** | **95% confidence interval** |
| --- | --- | --- | --- | --- |
| Bone | 0.735 | 0.443 - 0.874 | 0.802 | 0.583 – 0.906 |
| Cartilage | 0.710 | 0.392 – 0.862 | 0.670 | 0.307 – 0.843 |
| Rotator cuff muscles | 0.788 | 0.555 – 0.899 | 0.712 | 0.528 – 0.732 |
| Glenoid labrum | 0.613 | 0.187 – 0.816 | 0.567 | 0.419 – 0.639 |
| Deltoid muscle | 0.715 | 0.401 – 0.864 | 0.783 | 0.506 – 0.812 |
| Supraspinatus tendon | 0.745 | 0.464 – 0.879 | 0.691 | 0.351 – 0.853 |
| Infraspinatus tendon | 0.531 | 0.016 – 0.777 | 0.883 | 0.754 – 0.944 |
| Subscapularis tendon | 0.764 | 0.504 – 0.888 | 0.746 | 0.467 – 0.879 |
| Long head of biceps tendon | 0.910 | 0.806 – 0.958 | 0.946 | 0.883 – 0.975 |
| Acromioclavicular joint | 0.369 | 0.211 – 0.399 | 0.415 | 0.321 – 0.645 |
| Subcutaneous fat tissue | 0.643 | 0.250 – 0.830 | 0.630 | 0.408 – 0.681 |
| Overall | 0.695 | 0.641 - 0.741 | 0.595 | 0.522 - 0.656 |

Supplementary table 3

|  | **Conventional PROPELLER sequences**  **(ICC)** | **95% confidence interval** | **Post-processed PROPELLER sequences**  **using DL**  **(ICC)** | **95% confidence interval** |
| --- | --- | --- | --- | --- |
| Bone | 0.833 | 0.649 - 0.853 | 0.944 | 0.882 – 0.973 |
| Cartilage | 0.844 | 0.723 – 0.865 | 0.920 | 0.833 – 0.962 |
| Rotator cuff muscles | 0.874 | 0.736 – 0.940 | 0.912 | 0.728 – 0.924 |
| Glenoid labrum | 0.864 | 0.714 – 0.935 | 0.871 | 0.729 – 0.939 |
| Deltoid muscle | 0.823 | 0.583 – 0.905 | 0.794 | 0.568 – 0.902 |
| Supraspinatus tendon | 0.798 | 0.576 – 0.904 | 0.915 | 0.821 – 0.960 |
| Infraspinatus tendon | 0.894 | 0.777 – 0.949 | 0.912 | 0.815 – 0.958 |
| Subscapularis tendon | 0.910 | 0.806 – 0.958 | 0.946 | 0.883 – 0.975 |
| Long head of biceps tendon | 0.910 | 0.806 – 0.958 | 0.946 | 0.883 – 0.975 |
| Acromioclavicular joint | 0.809 | 0.600 – 0.909 | 0.915 | 0.821 – 0.960 |
| Subcutaneous fat tissue | 0.661 | 0.287 – 0.838 | 0.634 | 0.231 – 0.826 |
| Overall | 0.837 | 0.807 – 0.863 | 0.898 | 0.878 – 0.916 |

Supplementary table 4

Supplementary table 5

|  | **Conventional PROPELLER sequences**  **ICC** | **95% confidence interval** | **Post-processed PROPELLER sequences**  **using DL**  **(ICC)** | **95% confidence interval** |
| --- | --- | --- | --- | --- |
| Bone | 0.862 | 0.710 – 0.934 | 0.788 | 0.554 – 0.899 |
| Cartilage | 0.939 | 0.850 – 0.964 | 0.968 | 0.933 – 0.985 |
| Rotator cuff muscles | 0.829 | 0.642 – 0.919 | 0.882 | 0.751 – 0.944 |
| Glenoid labrum | 0.842 | 0.667 – 0.925 | 0.779 | 0.535 – 0.895 |
| Deltoid muscle | 0.669 | 0.411 – 0.827 | 0.801 | 0.583 – 0.905 |
| Supraspinatus tendon | 0.921 | 0.834 – 0.962 | 0.659 | 0.283 – 0.837 |
| Infraspinatus tendon | 0.873 | 0.732 – 0.939 | 0.918 | 0.827 – 0.961 |
| Subscapularis tendon | 0.931 | 0.854 – 0.967 | 0.896 | 0.781 – 0.950 |
| Long head of biceps tendon | 0.939 | 0.868 – 0.972 | 0.964 | 0.921 – 0.983 |
| Acromioclavicular joint | 0.682 | 0.332 – 0.849 | 0.787 | 0.547 – 0.900 |
| Subcutaneous fat tissue | 0.915 | 0.821 – 0.960 | 0.987 | 0.831 – 0.972 |
| Overall | 0.883 | 0.754 – 0.844 | 0.819 | 0.719 – 0.821 |

Supplementary table 6

|  | **Conventional PROPELLER sequences**  **(κ-value)** | **Post-processed PROPELLER sequences**  **using DL**  **(κ-value)** |
| --- | --- | --- |
| Bone | 0.725 | 0.821 |
| Cartilage | 0.741 | 0.841 |
| Rotator cuff muscles | 0.689 | 0.791 |
| Glenoid labrum | 0.821 | 0.860 |
| Deltoid muscle | 0.801 | 0.793 |
| Supraspinatus tendon | 0.907 | 0.815 |
| Infraspinatus tendon | 0.868 | 0.737 |
| Subscapularis tendon | 0.778 | 0.841 |
| Long head of biceps tendon | 0.758 | 0.664 |
| Acromioclavicular joint | 0.844 | 0.795 |
| Subacromial bursa | 0.852 | 0.892 |
| Overall | 0.840 | 0.842 |

Supplementary table 7

| **SNR** | | | | | |  |
| --- | --- | --- | --- | --- | --- | --- |
|  | | **Mean** | **n** | **Std. Deviation** | **Std. Error Mean** | **p-value** |
| Pair 1 | bone_std | 121,99 | 30 | 160,14 | 29,23 | 0.002 |
|  | bone_DL | 389,12 | 30 | 484,54 | 88,46 |  |
| Pair 2 | muscle_std | 232,65 | 30 | 185,13 | 33,80 | 0.002 |
|  | muscle_DL | 838,41 | 30 | 1034,48 | 188,86 |  |
| Pair 3 | fat_std | 116,08 | 30 | 98,43 | 17,97 | 0.003 |
|  | fat_DL | 404,98 | 30 | 532,00 | 97,12 |  |

| **Supplementary table 8**  **CNR** | | | | | |  |
| --- | --- | --- | --- | --- | --- | --- |
|  | | **Mean** | **n** | **Std. Deviation** | **Std. Error Mean** | **p-value** |
| Pair 1 | bone_muscle_std - bone_muscle_DL | -338,63 | 30 | 566,06 | 103,34 | 0.003 |
| Pair 2 | muscle_fat_std - cmuscle_fat_DL | -316,86 | 30 | 534,08 | 97,51 | 0.003 |
